# Protocol for quantitative evaluation of the impact of paracrine senescence on cellular reprogramming in cultured cells and mouse models

**DOI:** 10.1016/j.xpro.2023.102106

**Published:** 2023-02-07

**Authors:** Jérémy Chantrel, Cheng Chen, Jun Zhang, Han Li

**Affiliations:** 1 Cellular Plasticity in Age-related Pathologies, Department of Developmental & Stem Cell Biology, CNRS UMR 3738, Université Paris Cité, Institut Pasteur, 25 rue du Dr Roux, 75015 Paris, France; 2 Sorbonne Université, Collège Doctoral, 75005 Paris, France; 3 Télécom Paris, 19 Place marguerite Perey, 91120 Palaiseau, France

**Keywords:** Cell Biology, Cell culture, Microscopy, Model Organisms, Stem Cells

## Abstract

We present a protocol to evaluate the impact of senescence secretome on reprogramming to pluripotency using both cellular and mouse models. First, we describe the *in vitro* reprogramming procedure using conditioned medium derived from senescent cells. Next, to explore the impact of senescence on *in vivo* reprogramming, we detail the steps to identify senescent and reprogrammed cells in mouse skeletal muscle, followed by semi-automatic quantification. This protocol can be used to study the effect of paracrine senescence on cellular plasticity.

For complete details on the use and execution of this protocol, please refer to von Joest et al. (2022).^1^

## Highlights

• Protocol for studying the effect of paracrine senescence on cellular reprogramming
• Assessment of *in vitro* reprogramming in cultured cells and *in vivo* reprogramming in mice
• Steps for co-staining senescent cells with cell-identity markers in tissue
• Semi-automatic quantification of SA-β-Gal+ senescent cells in tissue sections

## Abstract (editor highlights)

Publisher’s note: Undertaking any experimental protocol requires adherence to local institutional guidelines for laboratory safety and ethics.

## Graphical abstract

**Figure:**
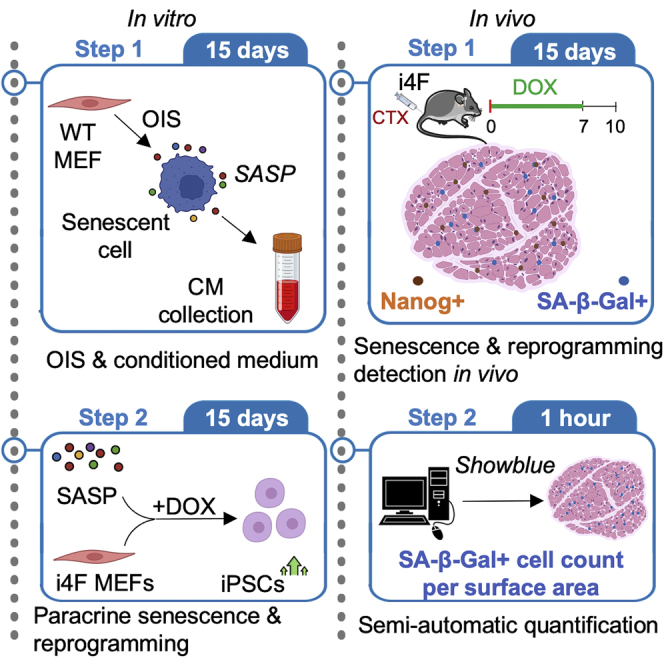

## Before you begin

Senescence regulates cellular plasticity in cell-autonomous and non-cell-autonomous manners. The senescence secretome, known as the senescence-associated secretory phenotype (SASP), is crucial for non-cell autonomous functions of senescence. Notably, SASP composition is stress- and cell-type dependent. To identify specific SASP factors important for promoting cellular plasticity, we use a reprogrammable mouse model (i4F), *Rosa26-rtTA*, and *TetO-OSKM*. Reprogramming can be induced by the addition of doxycycline (DOX). This protocol is divided into *in vitro* and *in vivo* sections. First, we describe the conditioned medium (CM) system to compare the effects of stress-dependent paracrine senescence on *in vitro* reprogramming. Second, to explore the impact of paracrine senescence on *in vivo* reprogramming, we detail the specific steps of the co-identification of senescence-associated β-galactosidase (SA-β-Gal+) and reprogramming cells (Nanog+) in the tibialis anterior (TA) muscle of reprogrammable mice. Previously, we’ve shown the combination of injury and DOX administration results in senescence induction and reprogramming of TAs.^2^ Finally, we describe a semi-automatic image analysis program for quantifying the number of senescent cells *in vivo*.

The *in vitro* part of the protocol can be applied to any cellular system of cell fate conversion, with some modifications to the CM supplementation step (see conditioned iPSC medium). The *in vivo* part of the protocol can be applied to any tissue from i4F mice, with some adjustments in SA-β-Gal staining incubation time, to animals of both sexes and of different ages, from adulthood to age. Moreover, this part of the protocol can facilitate the identification and quantification of senescent cells in physiological and pathological processes, beyond reprogramming.

The materials, reagents, concentrations, and equipment used in this protocol are described in detail below. All the cells used in this protocol are mouse embryonic fibroblasts (MEFs) collected at 13.5 embryonic stage following standard protocol.

Before beginning the protocol:

1. Derive MEFs from both WT and i4F mice.
2. Induce senescence in the WT MEFs.
3. Prepare the following solutions listed in materials and equipment.

### Institutional permissions

All procedures involving mice, including breeding and experiments, were performed according to the European Community guidelines and protocols approved by the ethics committee of the Institut Pasteur (CETEA).

## Key resources table

**Table:** 

REAGENT or RESOURCES	SOURCE	IDENTIFIER
**Antibodies**		

F4/80 (1:200)	Invitrogen	MA516624
Rabbit anti-Nanog (H22) (2 μg/mL)	Cambridge Research Biochemicals (CRB)	Custom-made
HRP-anti-rabbit (Ready for use, 200 μL/reaction)	Dako	K4003

**Experimental models: Cell lines**		

293T/17 (recommended range of passage: 1–7)	ATCC	ATCC-CRL-11268

**Recombinant DNA**		

Plasmid: pBabe-Ras	Gift from William Hahn (viral vectors)	Addgene 9051
Plasmid: pCL Eco	Naviaux et al.^3^	Addgene 12371
Plasmid: pBabe-PURO-EGFP(PIG)	Gift from Scott Lowe (Lowe Lab Plasmids)	Addgene 18751

**Experimental models: Organisms/strains**		

C57BL/6 WT mice (8 weeks old males)	Jackson Laboratory	000664
i4F mice (8 weeks old males, JCW; rtTA+/KI; OSKM+/Tg)	Gift from Manuel Serrano^4^	N/A

**Chemicals, peptides, and recombinant proteins**		

Dulbecco’s Modified Eagle Medium (DMEM)	Gibco	31966-021
Fetal bovine serum (FBS), previously inactivated at 56°C for 30 min in a water bath	Gibco	10270-106
KnockOut™ Serum Replacement (KSR)	Gibco	10828-028
Phosphate-buffered saline (PBS), without calcium, and without magnesium	Gibco	14190144
GlutaMAX™ 100×	Gibco	35050-061
Gelatin		
Non-essential amino acids 100×	Sigma-Aldrich	M7145
β-mercaptoethanol	Gibco	31350-040
Penicillin/streptomycin (Pen/Strep) 100×	Gibco	15140-122
Leukemia inhibitory factor (LIF)	Miltenyi Biotec	130-095
Doxycycline (DOX)	Sigma-Aldrich	9891
Fugene6	Promega	E2693
Polybrene (0.8 mg/mL stock solution)	ABP Biosciences	D025
Cardiotoxin	Merck Chimie SAS	217503
2-methylbutane	Sigma-Aldrich	320404-1L
Tragacanth	VWR PROLABO	24437.260
32% Paraformaldehyde (PFA)	Electron Microscopy Science	15714
50% Glutaraldehyde	Sigma	49629
Triton X-100	Sigma	9002-93-1
X-Gal	Euromedex	EU0012
Potassium hexacyanoferrate (iii) K_3_Fe(CN)_6_	Sigma	P8131
Potassium hexacyanoferrate (ii) K_4_Fe(CN)_6_	Sigma	P9387
MgCl_2_	Sigma	M8266
NP40	Sigma	I8896
Normal goat serum (NGS)	Thermo Fisher	10189722
Bovine serum albumin (BSA)	Sigma	A3608
Fast-red	Vector	H3403
Eukitt ® Quick-hardening mounting media	Sigma	03989

**Critical commercial assays**		

DAB staining kit	Agilent Technologies France	K346811-2
Alkaline phosphatase activity assay	Sigma	AB0300

**Software and algorithms**		

Fiji 2.9.0	Schindelin et al.^5^	https://imagej.net/software/fiji/
GraphPad Prism 9.4.1	GraphPad Software, San Diego, California USA	www.graphpad.com
Python 3.8	Python Software Foundation	https://www.python.org/
Anaconda Software Distribution	Anaconda Inc.	https://www.anaconda.com/products/distribution
PyCharm 2022.3.1	JetBrains	https://www.jetbrains.com/pycharm/

**Deposited data**		

The *Showblue* code	This paper	https://github.com/CroixJeremy2/Showblue or https://zenodo.org/record/7528943

**Other**		

P100 plates	Corning	353003
6-well plates	Corning	353046
Superfrost plus slides	Fisher Scientific	10149870
DAKO pen	Agilent Technologies France	S200230-2
Scanning microscope vs120	Olympus	VS120
Inverted cell culture microscope	Olympus	CKX41
0.20 μm sterile filter	Clearline	146560
0.45 μm sterile filter	Clearline	146561
Syringe	BD Medical	10 mL: 309110 20 mL: 300865 50 mL: 300613
Cork	Dutscher Dominique	764020
Ultracentrifuge	Beckman Coulter	Optima XPN-80 (SW32Ti rotor)

**CRITICAL:** DAB is a Carcinogenic, mutagenic and reprotoxic (CMR) chemical. It is toxic to reproduction. Always wear proper protective equipment and use it only in a fume hood with filter tips. DAB wastes need to be collected and disposed according to institutional regulations.

## Materials and equipment

### *In vitro* cell culture media

**Table:** For culture senescent MEFs: MEF medium

Reagent	Stock concentration	Final concentration	Amount
Dulbecco’s Modified Eagle Medium (DMEM)	100%	90.50%	500 mL
Fetal Bovine Serum (FBS)	100%	9.05%	50 mL
Penicillin/Streptomycin (Pen/Strep)	10,000 U/mL	45.25 U/mL (0.45%)	2.5 mL
**Total**	N/A	N/A	**552.5 mL**

Can be stored at 4°C up to 3 months.

**Table:** For collecting senescent conditioned medium: conditioned MEF medium

Reagent	Stock concentration	Final concentration	Amount
DMEM	100%	90.50%	500 mL
KSR (KnockOut™ Serum Replacement)	100%	9.05%	50 mL
Pen/Strep	10,000 U/mL	45.25 U/mL (0.45%)	2.5 mL
**Total**	N/A	N/A	**552.5 mL**

Can be stored at −20°C up to 1 month.

**Table:** For reprogramming: iPSC medium

Reagent	Stock concentration	Final concentration	Amount
DMEM	100%	82.47%	500 mL
KSR (KnockOut™ Serum Replacement)	100%	14.85%	90 mL
Pen/Strep	10,000 U/mL	49.48 U/mL (0.49%)	3 mL
Non-essential amino acids	100×	1× (0.99%)	6 mL
GlutaMAX™	100×	1× (0.99%)	6 mL
β-mercapto ethanol	50 mM	98.97 μM (0.20%)	1.2 mL
Leukemia Inhibitory Factor (LIF)	1.0 × 10^7^ U/mL	989.67 U/mL (0.01%)	60 μL
**Total**	N/A	N/A	**606.26 mL**

Can be stored at 4°C up to 2 months.

**Table:** For reprogramming with senescent conditioned medium: conditioned iPSC medium

Reagent	Stock concentration	Final concentration	Amount for 10 mL
Senescent conditioned medium	100%	82.79%	8.279 mL
KSR (KnockOut™ Serum Replacement)	100%	15%	1.5 mL
Non-essential amino acids	100×	1× (1%)	100 μL
GlutaMAX™	100×	1× (1%)	100 μL
β-mercapto ethanol	50 mM	2 μM (0.20%)	20 μL
LIF	1.0 × 10^7^ U/mL	1000 U/mL (0.01%)	1 μL
**Total**	N/A	N/A	**10 mL**

Must be prepared freshly: reconstitute senescent conditioned medium with additional KSR and other reagents listed above before use.

DOX solution stock: 1 mg/mL in H_2_O, stored at −20°C for up to two years.

### Senescent cell detection: SA-β-gal staining

**Table:** SA-β-Gal staining fixation buffer

Reagent	Stock concentration	Final concentration	Amount
Paraformaldehyde (PFA)	32%	2%	20 mL
Glutaraldehyde	50%	0.2%	1.28 mL
PBS	N/A	N/A	298.72 mL
**Total**			**320 mL**

The fixation solution can be stored at 4°C up to 6 months.

**Table:** SA-β-Gal staining buffer

Reagent	Stock concentration	Final concentration	Amount
K_3_Fe(CN)_6_	100 mM	4 mM	400 μL
K_4_Fe(CN)_6_	100 mM	4 mM	400 μL
MgCl_2_ (0.20 μm filtered)	1 M	2 mM	20 μL
X-Gal solution	50 mg/mL	0.4 mg/mL	80 μL
NP40	100%	0.1%	10 μL
PBS (pH = 5.5)	N/A	N/A	9.09 mL
**Total**			**10 mL**

Must be prepared freshly on the day required.

**CRITICAL:** PFA and Glutaraldehyde are hazardous. Always wear proper protective equipment and use it only in a fume hood. PFA wastes need to be collected and disposed according to institutional regulations.

**Table:** Immunohistochemical blocking solution

Reagent	Stock concentration	Final concentration	Amount
Triton X-100	10%	0.3%	300 μL
Normal goat serum	100%	10%	1 mL
BSA	10%	0.2%	200 μL
PBS	N/A	N/A	8.5 mL
Total			10 mL

Blocking solutions must be prepared freshly.

## Step-by-step method details

### PART I: *In vitro* reprogramming using senescence conditioned medium

#### Senescent cells generation via oncogene overexpression: Oncogene-induced senescence (OIS)

**Timing: 9 days**

This section describes how to generate senescent cells via the OIS method.

1. Day 1:
  a. Seed HEK293T cells at 5 × 10^6^ cells/P100 plate in MEF medium overnight.
  b. Prepare the plasmid cocktails:
    i. 4 μg of pBabe-ras + 4 μg of pCLEco.
    ii. 4 μg of pBabe-PURO-EGFP (PIG) + 4 μg of pCLEco.
  c. For each plasmid cocktail, prepare a mix of 24 μL of Fugene6 and 576 μL of DMEM (600 μL total volume), and incubate at room temperature (RT, 20°C–25°C) for 5 min.
  d. Add the plasmid cocktail into the DMEM + Fugene6 mix and incubate for 45 min at RT.
  e. Add the DNA/Fugene6 mix to the HEK293T cells.
    ***Note:*** Do not remove the medium from the culture plate, simply add the Fugene6/DNA mix to the medium dropwise with the P1000 pipette, distributing it around the plate.
  f. The same day, thaw one vial of the primary MEFs, and plate all of them onto one P100 plate.

2. Day 2:
  a. Replace the HEK293T cells medium with 10 mL of fresh MEF medium.
  b. Seed MEFs at 5 × 10^5^ cells/P100 plate for infection in MEF medium.
3. Day 3:
  a. Collect the medium from transfected HEK293T cells and replace with 10 mL of fresh MEF medium.
  b. Centrifuge each medium separately at 2,000 *g* for 5 min at RT to remove cell debris.
  c. Filter the supernatants with 0.45 μm filters separately.
  d. Add Polybrene (8 μg/mL final concentration).
  e. Repeat the infection one more time after 8–10 h.
4. **Day 4:** Remove the medium, and refresh with 10 mL MEF medium.
5. Day 5:
  a. Trypsinize the cells from each plate and reseed them at the 5 × 10^5^ cells/P100 plate with MEF medium containing Puromycin (1 μg/mL).
  b. Plate non-infected WT MEFs at the same condition as the negative control for Puromycin selection.
6. **Day 7:** Refresh the medium with MEF medium containing 1 μg/mL Puromycin.
7. **Day 9:** When there is no MEFs visible from the negative control plate (usually after 96 h), remove the medium and replace with fresh conditioned MEF medium without Puromycin for conditioned medium collection.

**Figure 1:**
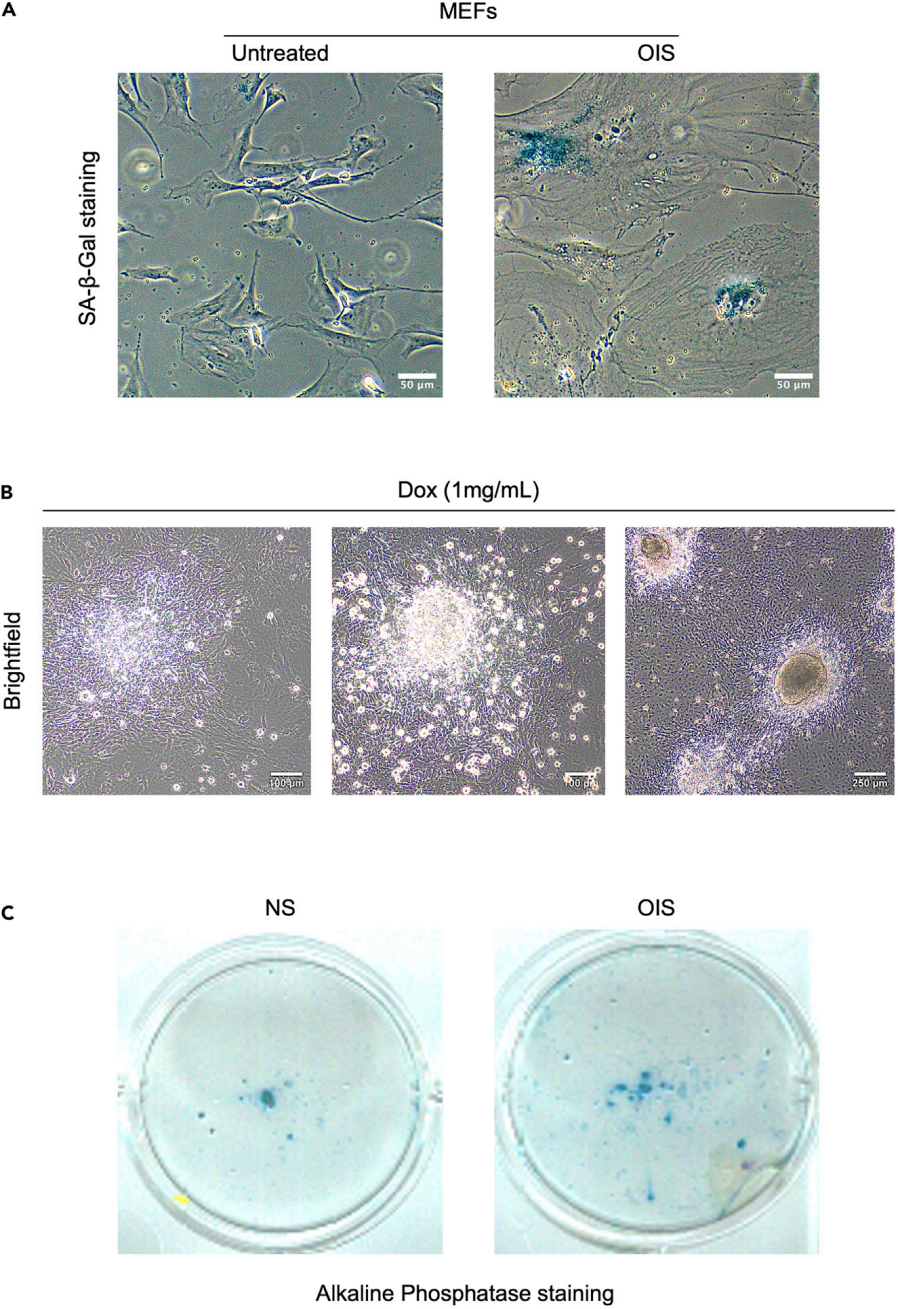
Senescent MEFs and *in vitro* reprogramming (A) SA-β-Gal staining of MEFs. Left: Control PIG infected MEFs. Right: oncogene (ras) induced senescent (OIS) MEFs. (B) Pictures of representative images of reprogrammable MEFs upon Doxycycline treatment (1 mg/mL). Left: pre-iPSC foci characterized by converging cells with bright edges (D4). Middle: Emerging iPSC colony characterized by bright spherical cells in the center (D8). Right: Full iPSC colonies characterized by a clear-reflective edge separation from the surrounding non-reprogrammed cells. (C) iPSCs colonies stained by the Alkaline phosphatase staining kit (Sigma, AB0300). Left: CM: non-senescent WT MEFs. Right: CM: IR-senescent WT MEFs.

***Note:*** At this point, if you perform the SA-β-gal assay, OIS-senescent MEFs will be stained in blue while non-senescent MEFs will not (Figure 1A).

#### Collection of senescent conditioned medium

**Timing: 6 days**

This section describes how to collect the conditioned medium from senescent cells.

8. After two days in conditioned MEF medium, collect the culture medium from both conditions, and replenish fresh conditioned MEF medium for next rounds of collection.

***Note:*** Senescent conditioned medium can be collected three times (48 h interval).

***Note:*** Make sure to collect the conditioned medium from the non-senescent counterpart (PIG-infected plate), which should be used as the control for senescent conditioned medium.

9. For the collected culture medium, centrifuge for 5 min at 500 *g* at RT.
10. Filter the supernatant with a 0.20 μm sterile filter, and store the filtered medium in several aliquots at −20°C.

**Pause point:** Filtered conditional medium can be stored at −20°C for up to 1 month.

**CRITICAL:** Repeated freeze/thaw cycles could greatly diminish the effects of the conditional medium. Therefore, we recommend to aliquot conditional media in proper aliquoted volumes depending on the experimental requirements to avoid repeated freeze/thaw cycles.

11. If separation of the exosomes from the soluble fraction is desired:
  a. Centrifuge the conditioned medium at 12,000 *g* for 30 min at 4°C, and retain the supernatant and discard the pellet.
  b. Ultracentrifuge the supernatant at 100,000 *g* for 3 h at 4°C, and separate the supernatant from the pellet.
    ***Note:*** The supernatant contains the soluble fraction, and the pellet contains the exosomes.
  c. Store the soluble fraction at −20°C in aliquoted volumes.
  d. Wash once by resuspending the exosomes in 1 mL PBS.
  e. Ultracentrifuge at 100,000 *g* for 3 h at 4°C, discard the supernatant, and keep the pellet.
  f. Resuspend the pellet in 20–50 μL PBS and store it at −20°C.
    **Pause point:** Exosomes in PBS can be stored for up to 1 month at −20°C, or alternatively for up to 1 year at −80°C, avoiding repeated freeze/thaw cycles.

#### Induction of reprogramming via senescent conditioned medium

**Timing: 10–14 days**

This section describes how to induce reprogramming in i4F MEFs using senescent conditioned medium.

12. Seed i4F MEFs at a density of 5.2 × 10^2^ cells/cm^2^ (that is*,* 5.0 × 10^3^ cells per well of a 6-well plate) in MEF medium.

***Note:*** The seeding density of the i4F MEFs is highly dependent on their reprogramming capacity. Therefore, we recommend testing the reprogramming efficiency of individual i4F MEF before this experiment.

13. The following day, remove MEF medium, and replace it with conditioned iPSC medium freshly supplemented with DOX (1 μg/mL).

**CRITICAL:** DOX must be added freshly to iPSC medium because its stability rapidly decreases at 37°C. We recommend changing iPSC medium supplemented with DOX every two days maximum.

14. Change the conditioned iPSC medium supplemented with DOX (1 μg/mL) every day.
15. After 3 days, change the conditioned iPSC medium to normal iPSC medium freshly supplemented with DOX (1 μg/mL).
16. Renew the medium every 2 days.

***Note:*** It takes approximately 10–14 days to generate iPSC colonies using a reprogrammable MEFs system. The experimental length and period of conditioned iPSC medium treatment may vary depending on the reprogramming system implemented. DOX concentration may also be an important factor in reprogramming efficiency for different cell types; thus, we recommend testing different DOX concentrations.

17. Once iPSC colonies become visible (Figure 1B), stain the plates with Alkaline Phosphatase staining kit following manufacturer’s recommendations.
  a. Bring both BCIP and NBT solutions at RT.
  b. In the meantime, remove the medium. Wash the plate once with PBS at RT.
  c. Fix cells with 4% PFA for 15 min at RT.
  d. Wash cells three times with PBS at RT, 5 min/wash.
  e. Mix equal volumes of the two component solutions (BCIP and NBT). The mixture should be used within 1 h.
  f. Add the mixed staining solution sufficient to cover the plate, such as 0.5 mL for one well of 6-well plate.
  g. Place the plates on a shaker at RT protected from light and agitate mildly to ensure that the staining solution covers the plate.

***Note:*** A bluish-purple staining will form within 15–30 min (Figure 1C).

### PART II: *In vivo* identification of senescent and pluripotent cells: IHC Co-staining with SA-β-gal staining

#### Tissue collection and procession

**Timing: 1 day**

This section describes how to generate cryosections from injured murine skeletal muscle.

18. 10 days prior of the sample collection, inject cardiotoxin (CTX) into tibialis anterior (TA) muscle of mice of both sex (2-month-old, C57/B6) to injury muscle as previously described.^6^

***Note:*** Make sure to inject PBS in the TA of the same mouse as non-injured negative control.

19. Collect TAs at 10 days post-injury:
  a. Place a small amount of tragacanth gum on a slice of cork.
  b. Extract both TA muscles as previously described.^7^
  c. To ensure the transverse sections, insert the distal tendon of the TA muscle (1/4 part) into the tragacanth gum and freeze directly in liquid nitrogen cooled isopentane for 40 s as previously described.^7^
    ***Note:*** Make sure the TA muscle is in a perpendicular position and in the center of the cork.
    **Pause point:** The samples can be stored at −80°C up to 2 years.
  d. Generate 10 μm cryosections as previously described.^7^

#### Senescence-associated β-galactosidase staining

**Timing: 48 h**

This section describes how to perform the SA-β-Gal assay on muscle cryosections.

20. Allow evaporation of tissue freezing medium for 30 min at room temperature (RT, 20°C–25°C).
21. To separate the control and save the antibody (for the immunostaining step), before placing the slides in solution, circle the section with a hydrophobic DAKO pen for IHC staining (Figure 2A).

**Figure 2:**
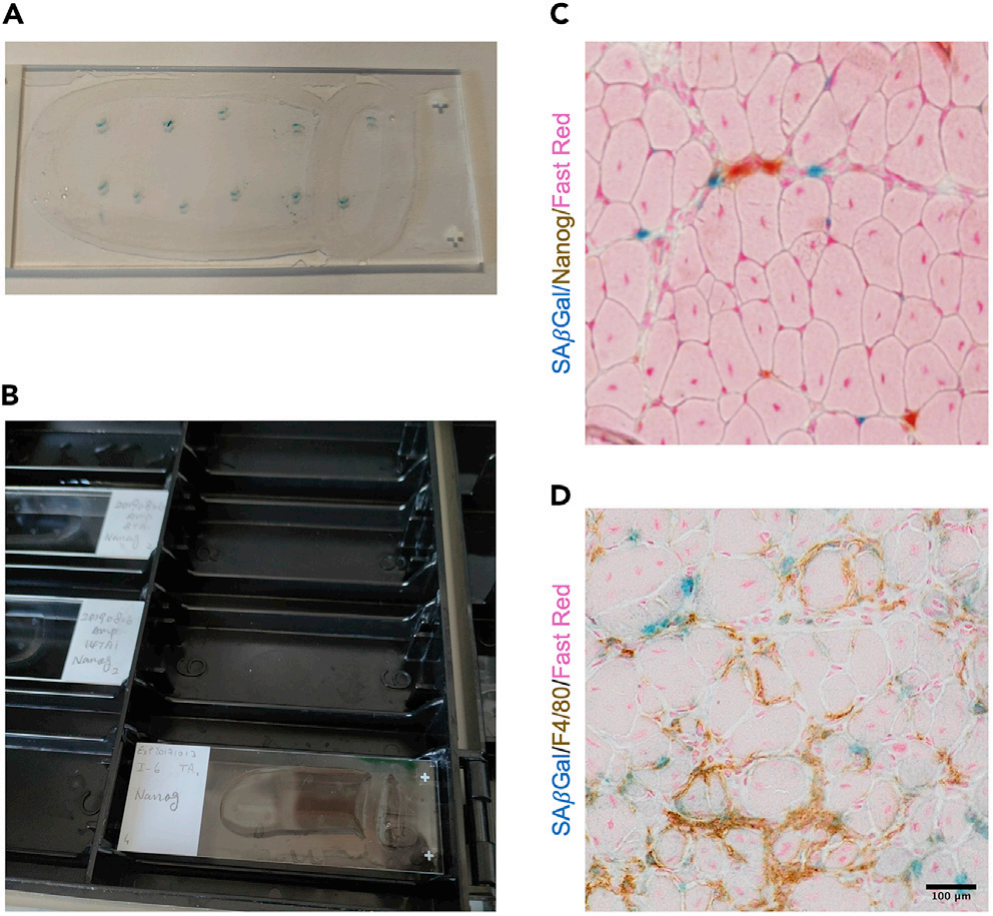
The co-staining of SA-β-Gal and Nanog (IHC) (A) Circle the sections and SA-β-Gal staining. (B) Incubation in the dark box. (C and D) Representative of the co-staining. Blue dot is SA-β-Gal positive cell and brown color stained the Nanog (C) and F4/80 (D) positive cell.
22. Fix the sections for 15 min in SA-β-Gal staining fixation buffer at RT.

**CRITICAL:** SA-β-Gal staining fixation buffer cannot be replaced by 4% PFA, and cannot be longer than 15 min.

23. Quickly wash (<1 min/wash) the slides three times with PBS.
24. Incubate the slides in the SA-β-Gal staining buffer at 37°C protected from light for 48 h.

***Note:*** Cover the container with Aluminum foil to prevent evaporation.

***Note:*** For quantitative comparisons, all sections must be stained with the same batch of SA-β-Gal staining buffer to avoid inter-assay variation.

**CRITICAL:** pH level is critical for the staining solution (pH = 5.5).

25. Refresh the staining solution after 24 h.
26. Check the slices using an inverted cell culture microscope (OLYMPUS CKX41). Once the blue color is visible (Figure 2A), quickly wash the slices three times with PBS.

***Note:*** The staining duration is tissue section dependent (tissue origin and section thickness). It needs to be optimized first and should be kept consistent for the samples from the same experiment. For example, for the muscle samples, we incubate 10 μm sections for 48 h. For other tissues, the staining need to be optimized further. In general, thicker sections need shorter incubation time than thinner sections. For example, sections from kidney and lung need shorter incubation time than muscle.

#### Immunohistochemistry staining (IHC)

**Timing: 24 h**

This section describes how to perform IHC staining targeting Nanog (or F4/80) on muscle cryosections. These procedures are performed following SA-β-Gal staining.

27. Quickly wash the slices three times with PBS and put the slides in 4% PFA in PBS for post-fixation for 15 min at RT.

**CRITICAL:** Post-fixation cannot be longer than 15 min.

28. Quickly wash the slides three times with PBS and place them in a humid dark chamber (Figure 2B).
29. Add 200 μL blocking buffer in every circle (labeled at step 21) for at least 30 min at RT. Prepare Nanog 1^st^ antibody at 1:200 in blocking buffer.

***Note:*** The dilution of 1^st^ antibody depends on different antibody.

30. Remove the blocking buffer carefully. Add 200 μL 1^st^ antibody to every circle of all the slides. For the control sections, add 200 μL blocking buffer.
31. Incubate the slides overnight at 4°C in a humid dark chamber.
32. Put the humid dark chamber at RT for 20–30 min and quickly wash with PBST (PBS +0.3% Triton X-100) three times.
33. Add 3% H_2_O_2_ in PBS for 20 min at RT.
34. Add 2^nd^ antibody (Dako, ready for use, K4003) directly for 90 min at RT in the humid dark chamber.

***Note:*** The duration of the 2^nd^ antibody incubation depends on different antibody.

35. Prepare the DAB solution (add 20 μL substrate in 1 mL buffer according to manufacturer’s instructions).

***Note:*** protect the DAB solution from the light.

36. Wash three times with PBST (PBS + 0.3% Triton X-100), 5 min/wash.
37. Develop with DAB for 1 min at RT, quickly wash the slides in Milli-Q water three times.
38. Counterstain with Fast-red for 10 min at RT.
39. Wash in tap water for 10 min.
40. Dehydrate in 95% ethanol for 10 min, followed by 100% ethanol baths twice, 5 min each.
41. Mount with Eukitt following manufacturer’s recommendations.
42. Evaporate in the hood overnight and scan the slides using a scanning microscope OLYMPUS vs120.

**CRITICAL:** All the primary antibodies dilution and the duration of DAB staining needs to be optimized first.

***Note:*** This protocol can be used for other antibodies, such as F4/80.

### PART III: Semi-automatic quantification of senescent cells based on SA-β-Gal staining.

The entire procedure is explained in Methods video S1 (or on YouTube with subtitles: https://www.youtube.com/watch?v=BHaThFfpkRY) to guide you in running *Showblue*.

Methods video S1. How to run Showblue, related to all the steps in PART III A YouTube video containing subtitles is also available following that link: https://www.youtube.com/watch?v=BHaThFfpkRY.

#### Installation of *Showblue* and its dependencies

**Timing: 30 min**

This section describes how to install Anaconda, Pycharm, Library cv2 and numpy packages, and *Showblue* on your computer (macOS or Windows).

43. Install these two programs on your computer:
  a. Anaconda.
  b. PyCharm (community version, optional).

***Note:*** Anaconda allows the installation of Python and the libraries required to implement the *Showblue* code. PyCharm allows to edit the *Showblue* code.

44. Install dependencies by typing these two lines of code in the Terminal (macOS) or in Anaconda Prompt (Windows).
  a. pip install opencv-python.
  b. conda install numpy.

***Note:*** Several Python libraries are required to support *Showblue*: Library cv2 (4.4.0.44) and numpy (1.18.5) (compatible with the newest version).

45. Download *Showblue* at https://github.com/CroixJeremy2/Showblue (or at https://zenodo.org/record/7528943) and decompress the .zip folder.

**Figure 3:**
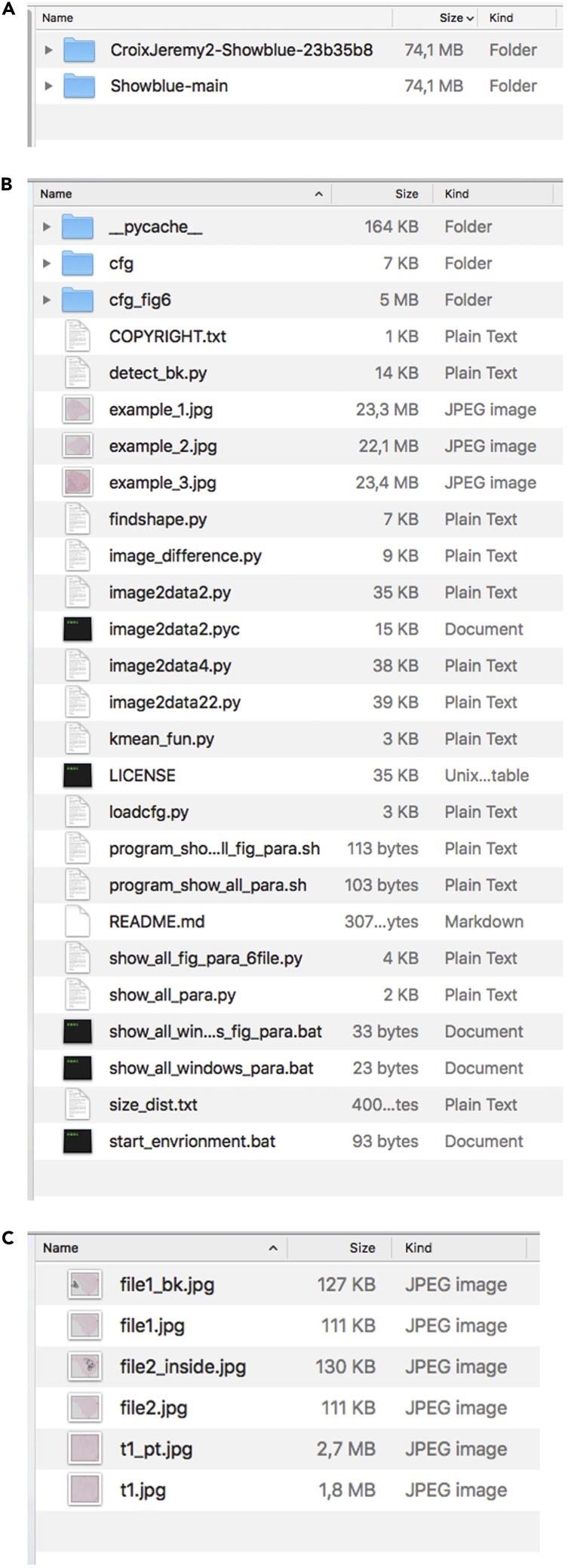
Quantification folder for SA-β-Gal staining (A) Folder content. (B) Folder (cfg_fig6) for reference picture. (C) List of sample pictures.

***Note:*** The decompressed folder named “Showblue-main” (or “CroixJeremy2-Showblue-23b35b8” if downloaded via Zenodo) contains all the files displayed in Figures 3A–3C.

***Note:*** on Windows, the content of “start_environment.bat” must be modified as “%windir%\System32\cmd.exe "/K" **[the path of anaconda folder]\**Scripts\activate.bat **[the path of anaconda folder]**”**.** For example, if the path to Anaconda is “**D:\programs\anaconda**”, then the content of “start_environment.bat” must be modified as “%windir%\System32\cmd.exe"/K" **D:\programs\anaconda**\Scripts\activate.bat **D:\programs\anaconda**”.

#### Preparation of test sample images

**Timing: 30 min**

This section describes how to prepare the test sample images in order to set up *Showblue* parameters.

**Figure 4:**
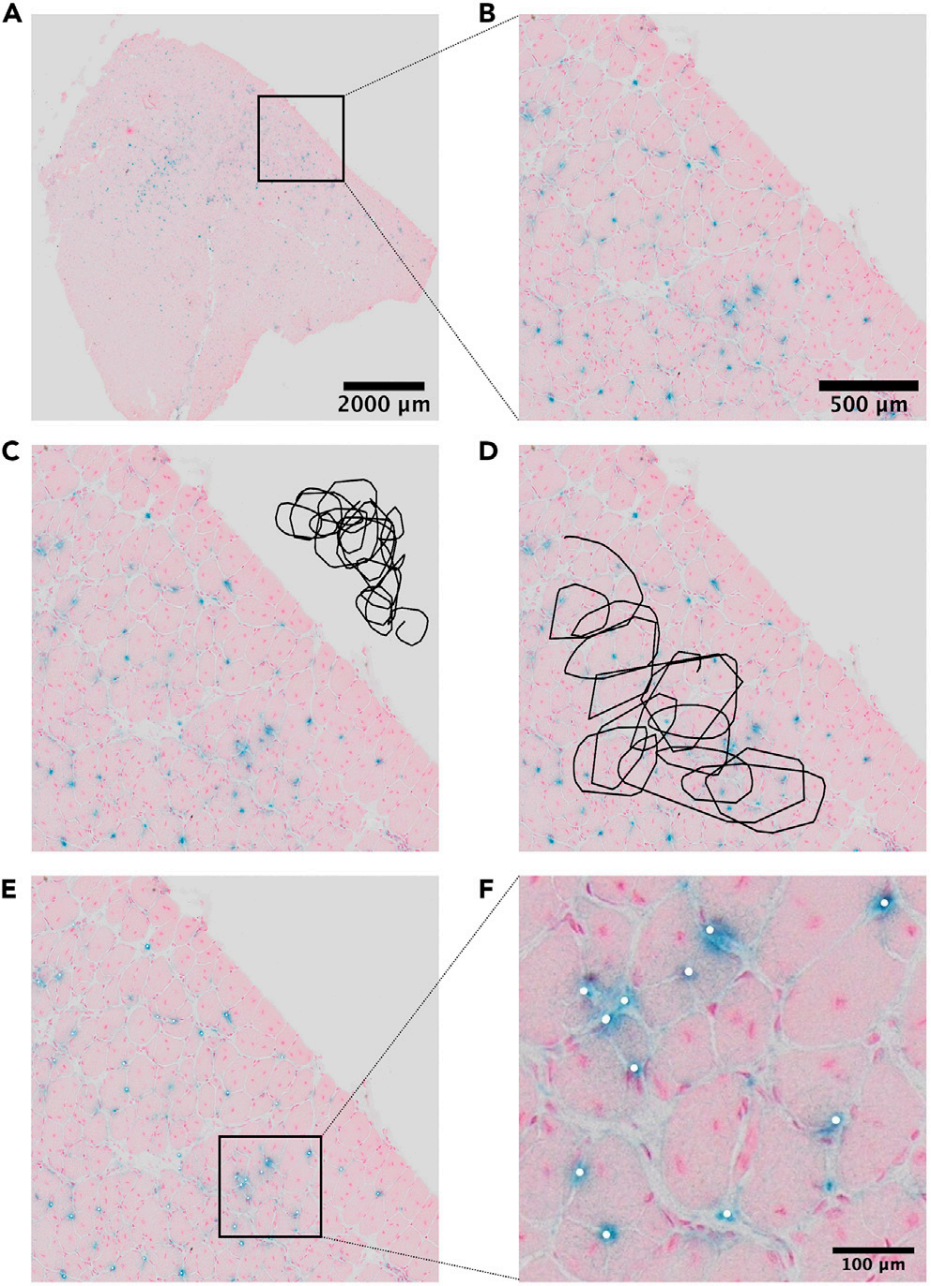
Setting up the reference picture (A) Selected picture. (B) Selected part of the picture. (C) file1_bk for labeling the blank part of the picture. (D) file2_inside for labeling the inside part of the picture. (E) t1_pt for pointing out the positive cells. (F) Zoom in for (E).
47. Crop a part of the image next to the edge (Figure 4B), and save three copies named “file1.jpg”, “file2.jpg”, and “t1.jpg” respectively in the folder named “cfg_fig6” (Figure 3B).
48. Label the “blank area” by drawing black scribbles outside of the muscle section via an image editor software (e.g., Fiji, Paint, or Paint X) on “file1.jpg” and save the resulting image as “file1_bk.jpg” in the “cfg_fig6” folder (Figure 4C).
49. Label the “inside area” by drawing black scribbles inside the muscle section on “file2.jpg” and save it as “file2_inside.jpg” (Figure 4D).
50. Label SA-β-Gal+ cells by drawing white dots on top of them on “t1.jpg” and save it as “t1_pt.jpg” (Figures 4E and 4F).

***Note:*** For samples from different experiments or with different colors, it is necessary to reset the references. A representative image should be selected as a reference. Pre-run the program to set up all preferences following steps 51–53. If there are few false positive or false negative detections, the program quantifies all pictures with the same preferences. Otherwise, the preferences must be adjusted manually, or the reference picture should be changed.

46. Choose one representative image from your samples (Figure 4A).

#### Pre-run *Showblue* to set up automatically the parameters, or manually set up the parameters.

**Timing: 10 min–45 min**

This section describes how to pre-run *Showblue* on test sample images (prepared in steps 46–50) to set up automatically *Showblue* parameters. In addition, this section describes how to adjust manually the parameters if automatic settings are not satisfactory.

51. Pre-run *Showblue* with the annotated test sample images:
  a. On macOS, right-click on the “showblue-main” folder and select “new terminal at folder”. The Terminal will appear, drag the file named **show_all_fig_para.sh** into the Terminal, and press enter.
  b. On Windows, double click on the **start_envrionment.bat** file. A window will open, drag into it the file named **show_all_windows_fig_para.bat**, and press enter.

***Note:****Showblue* will quantify the images automatically. When the quantification is finished, a new file named “data.txt” and a new folder named “process” will be generated in the “showblue-main” folder.

***Note:****Showblue* will also set up all text parameters (*cfg_bk.txt*, *cfg_in.txt*, and *cfg_pt.txt*) in the “cfg” folder automatically according to the labeled images in the “cfg_fig6” folder. If the results are not satisfactory, the text parameters can be further adjusted manually (step 53).

**Figure 5:**
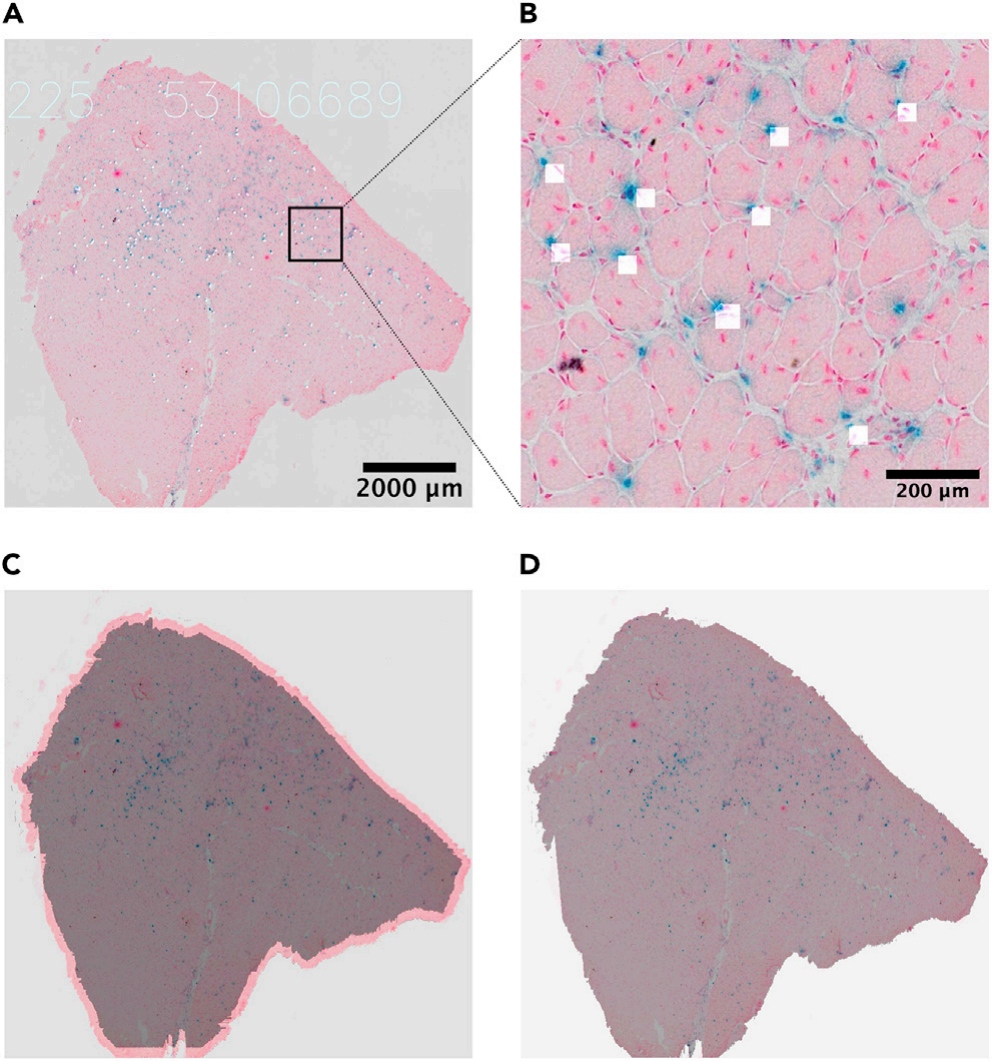
Quantification results (A) Files labeled “blue” display the number of SA-β-Gal+ cells (top left) and the total surface area (top right). (B) Zoom in of (A). (C) Files labeled “bound” display the edge detection of the muscle section. (D) Files labeled hull display the surface area that is quantified by *Showblue*.

***Note:*** The image labeled “blue” displays the number of detected SA-β-Gal+ cells (top left) and the pixels^2^ surface area of the muscle section (top right) (Figures 5A and 5B). The image labeled “bound” displays the muscle section boundaries that are excluded from the analysis (Figure 5C). And the image labeled “hull” displays the area that is analyzed (Figure 5D).

52. Check the quantification results in the “process” folder (Figure 5).

53. Configure the *Showblue* settings manually if automatic settings are not satisfactory, or if you want to further optimize the detection. The text parameters are located in the folder named “cfg”:
  a. cfg_alpha.txt: the “alpha value” is used to adjust the sensitivity of tissue boundary detection. By default, the alpha value is set to 1. The larger the alpha value, the higher the sensitivity.
  b. cfg_bk.txt: it describes the “mean color value ± the standard deviations (SD)” of the points outside the tissue boundary (background).
  c. cfg_in.txt: it describes the “mean color value ± SD” of all the points inside the tissue boundary.
  d. cfg_pt.txt: it describes the “mean color value ± SD” of the points of interest inside the tissue boundary.
    ***Note:*** The color value is defined using an RGB color model. Each of the primary additive colors of red, green, and blue is assigned a value in the range of 0–255.
  e. cfg_shape.txt: the “shape value” is to remove colored cells whose shape is not of interest. The value is set ranging from 0–1. If the value is 0, all colored items are counted as cells, including dye contaminations. When the value approaches 1, the selected cells are closer to the round shape and the counting sensitivity is low.
    ***Note:*** Usually, “cfg_alpha.txt” and “cfg_shape.txt” are the two main parameters that need to be adjusted according to the detection results after running *Showblue*.

#### Run *Showblue* to quantify all images at the same time

**Timing: depends on the computer power, and on the numbers and size of the images**

This section describes how to run *Showblue* on a batch of images.

54. Copy and paste all the images that need to be quantified into the “showblue-main” folder.
55. Run *Showblue*:
  a. On macOS, right-click on the “showblue-main” folder and select “new terminal at folder”. The Terminal will appear, drag the file named **show_all_fig_para.sh** in the Terminal, press enter, and let the computer work automatically.
  b. On Windows, double click on the **start_envrionment.bat** file. A window will open, drag into it the file named **show_all_windows_para.bat**, press enter, and let the computer work automatically.

***Note:*** depending on the computer power, and the number and size of images that need to be quantified, this step can vary considerably over time.

56. Check all the quantification results in the “process” folder as described in step_52 (Figures 5A–5D).
57. Import the “data.txt” file into a spreadsheet (e.g., Microsoft Excel) for further analysis.

***Note:*** Lines in the data.txt are formatted in the following order: [image name]: [counted points]: [pixel area]: [x-pixel]: [y-pixel]: [ratio of the size of the tissue to the image]: [ratio of painted area to the cell].

***Note:*** [pixel area] = [x-pixel] × [y-pixel]

58. Calculate the results with this equation:

$$SA-\beta -Gal+cells\;/\;unit\;area=\left[counted\;points\right]\;/\;\left[pixel\;area\right]\times 10^{6}$$

## Expected outcomes

The outcome of PART I is a reliable and consistent collection of senescent MEF conditional medium to study the effect of SASP on cellular reprogramming *in vitro*.^1^ The composition of the SASP is highly heterogeneous and can have a distinct effect on reprogramming efficiency. Therefore, it is crucial to understand how SASP composition affects different processes and how cellular reprogramming is initiated in a paracrine manner.

Here, we provide a feasible protocol for identifying and investigating the potential molecular mechanisms underlying these two processes. A successful and efficient reprogramming is characterized by the formation of ∼20 iPSC colonies per well of a 6-well plate within 14 days (Figure 1C) and quantified using Fiji.

Co-staining of SA-β-Gal with other markers in tissue samples has been difficult owing to incompatible fixation and staining conditions. Here in PART II, we present a robust protocol to simultaneously identify senescent (SA-β-Gal^+^) and reprogrammed (Nanog^+^) cells in the same TA muscle sample collected from reprogrammable mice. Successful co-staining is shown in Figure 2C, with SA-β-Gal staining in blue and IHC staining in brown. No signals were detected in the negative control (non-injured muscle for SA-β-Gal and the section without 1^st^ antibody for Nanog). This protocol can be used to determine the identity of senescent cells *in vivo*, when combined with cell identity markers. For example, macrophages have an increased lysosomal content and can be positive for SA-β-Gal staining. To determine whether SA-β-Gal^+^ cells were macrophages, we co-stained for SA-β-Gal and F4/80, a macrophage marker (Figure 2D). Interestingly, although many cells were positive for both markers, we also found that the cells were only positive for SA-β-Gal or F4/80, suggesting a heterogeneous senescent population (Figure 2D).

Quantifying SA-β-Gal-positive cells *in vivo* is time consuming and subject to user experience. We present a semi-automatic plug-in to facilitate this process. The false-positive or false-negative rates should not exceed 10% (Figure 5B), and calculation of the area should be performed automatically (Figure 5D). Although this program does not accurately count absolute numbers, the relative numbers are reproducible. Therefore, it is crucial to quantify samples from different groups simultaneously. It usually takes 1–2 h to set up the preferences and an hour to collect data with this program. Therefore, it provides an efficient and consistent alternative to the conventional manual counting methods.

## Quantification and statistical analysis

Quantification and statistical analysis should be performed by a blinded operator to avoid bias. Statistical analyses were performed using the GraphPad Prism v9 software.

## Limitations

The protocol described here aims to study the impact of paracrine senescence on cellular plasticity in the context of cellular reprogramming to pluripotency. Therefore, a reprogrammable mouse model and an MEF system are used to allow robust and reliable reprogramming induction. The link between senescence and plasticity has also been demonstrated in other physiological and pathological conditions. Therefore, the *in vitro* conditioned medium method can be modified to study how non-cell- autonomous senescence affects cellular plasticity beyond the reprogramming context. The method used to identify and quantify senescent cells can be applied to characterize *in vivo* senescence.

For *in vitro* experiments, the protocol is performed using conditioned medium derived from senescent MEFs only. Paracrine senescence is heterogeneous, which is stress- and cell-type dependent^8^; thus, conditioned medium from other cell types could be different from MEFs.

It is possible that senescent conditioned medium from certain cell types might prevent reprogramming. We encourage you to report such observations and studies to better understand the link between senescence and cellular reprogramming and plasticity.

*In vitro* reprogramming is performed using i4F MEFs only. Reprogramming kinetic and efficiency can vary greatly depending on the cell type and reprogramming methods. Therefore, the initial seeding density of the reprogramming cells, DOX concentration, and treatment length of the conditioned iPSCs medium should be determined first.

To ensure accurate quantification, well-preserved tissue sections and high-quality of the SA-β-Gal staining are crucial. In addition, only the samples processed, stained, and analyzed in the same patch can be compared.

## Troubleshooting

### Problem 1

You don’t obtain any iPSCs colony after applying conditioned medium on i4F MEFs (step 16, Figure 1B).

### Potential solution

Make sure the conditioned medium has not undergone more than one freeze/thaw cycle. Precipitates can form and alter conditional media composition, resulting in a poor reprogramming efficiency. We recommend to aliquot your conditional medium in small volumes. Moreover, the initial seeding density of the reprogramming cells might be too low. The reprogramming efficiency might vary significantly from one embryo to another, and by using different reprogramming systems. Therefore, the seeding density should be determined prior.

### Problem 2

You don’t see the blue developed after 48 h SA-β-Gal (step 26, Figure 2A).

### Potential solution

As described above, make sure pH is 5.5 for the PBS and all the other components for the staining solution are clean without contaminations. For the different tissue and the thickness of the sections, the incubation time is different. If the staining doesn’t work, the incubation time should be optimized. It is crucial to include positive and negative control for each experiment.

### Problem 3

Error for installation of library cv2 (step 44).

### Potential solution

Sometimes, there is a problem in installing cv2 with the error information as “Could not build wheels for opencv-python which use PEP 517 and cannot be installed directly”. In such a case, it is necessary to run the command “pip install --upgrade pip setuptools wheel” before installing of cv2.

## Resource availability

### Lead contact

Further information and requests for resources and reagents should be directed to and will be fulfilled by the lead contact, Han Li (han.li@pasteur.fr).

### Materials availability

This study did not generate new unique reagents.

## Data Availability

The *Showblue* code generated during this study is available at https://github.com/CroixJeremy2/Showblue. An archived version of record is also available at https://zenodo.org/record/7528943 (https://doi.org/10.5281/zenodo.7528943).
